# Interhemispheric Pathways Are Important for Motor Outcome in Individuals with Chronic and Severe Upper Limb Impairment Post Stroke

**DOI:** 10.1155/2017/4281532

**Published:** 2017-11-16

**Authors:** Kathryn S. Hayward, Jason L. Neva, Cameron S. Mang, Sue Peters, Katie P. Wadden, Jennifer K. Ferris, Lara A. Boyd

**Affiliations:** ^1^Department of Physical Therapy, University of British Columbia, Vancouver, BC, Canada V6T 1Z3; ^2^Stroke Division, Florey Institute of Neuroscience and Mental Health, University of Melbourne, Melbourne, VIC 3084, Australia; ^3^NHMRC Centre of Research Excellence in Stroke Rehabilitation and Brain Recovery, Melbourne, VIC 3084, Australia; ^4^Graduate Program in Rehabilitation Sciences, Faculty of Medicine, University of British Columbia, Vancouver, BC, Canada V6T 1Z3; ^5^Djavad Mowafaghian Centre for Brain Health, University of British Columbia, Vancouver, BC, Canada V6T 1Z3

## Abstract

**Background:**

Severity of arm impairment alone does not explain motor outcomes in people with severe impairment post stroke.

**Objective:**

Define the contribution of brain biomarkers to upper limb motor outcomes in people with severe arm impairment post stroke.

**Methods:**

Paretic arm impairment (Fugl-Meyer upper limb, FM-UL) and function (Wolf Motor Function Test rate, WMFT-rate) were measured in 15 individuals with severe (FM-UL ≤ 30/66) and 14 with mild–moderate (FM-UL > 40/66) impairment. Transcranial magnetic stimulation and diffusion weight imaging indexed structure and function of the corticospinal tract and corpus callosum. Separate models of the relationship between possible biomarkers and motor outcomes at a single chronic (≥6 months) time point post stroke were performed.

**Results:**

Age (Δ*R*^2^0.365, *p* = 0.017) and ipsilesional-transcallosal inhibition (Δ*R*^2^0.182, *p* = 0.048) explained a 54.7% (*p* = 0.009) variance in paretic WMFT-rate. Prefrontal corpus callous fractional anisotropy (PF-CC FA) alone explained 49.3% (*p* = 0.007) variance in FM-UL outcome. The same models did not explain significant variance in mild–moderate stroke. In the severe group, k-means cluster analysis of PF-CC FA distinguished two subgroups, separated by a clinically meaningful and significant difference in motor impairment (*p* = 0.049) and function (*p* = 0.006) outcomes.

**Conclusion:**

Corpus callosum function and structure were identified as possible biomarkers of motor outcome in people with chronic and severe arm impairment.

## 1. Introduction

Upper limb impairment post stroke is a devastating personal experience [1], and it remains challenging to prognosticate outcome [2]. To date, clinical measures administered early (<7 days post stroke) appear to be the best predictors of recovery of upper limb impairment and function [2]. However, there are important limitations to the use of clinical measures in people with severe upper limb impairment. First, a large amount of variability in motor outcome and recovery remains unexplained in people with severe upper limb impairment when only clinical measures are used during acute [2], subacute [3], and chronic [4] phases of recovery. Second, clinical measures do not provide information about the underlying neurobiology that may underpin outcome [5].

Increasing work suggests that brain biomarkers may be important to help better understand motor outcome and recovery of people with severe impairment after stroke [5–7]. A brain biomarker is an indicator of disease state that can be used as a measure of underlying molecular/cellular processes that may be difficult to measure directly in humans and could be used to understand outcome or predict recovery or treatment response [8]. While the ultimate goal for the field of stroke recovery is to identify brain biomarker/s in the early stages post stroke that can prognosticate potential for motor recovery [9], conducting longitudinal studies to this end is extremely time consuming and expensive. Cross-sectional studies, even in the chronic stage, are an important preliminary step that can identify possible brain biomarkers, which may be subsequently used to inform longitudinal studies [9].

The corticospinal tract (CST) has been identified as a biomarker of upper limb motor outcome in the chronic phase. Corticospinal tract indicators of poor outcome include (i) absence of an ipsilesional motor evoked potential (MEP) tested with transcranial magnetic stimulation (TMS) [7], (ii) poor integrity of CST streamlines indicated by low fractional anisotropy (FA) indexed with diffusion-weighted imaging (DW-MRI) [10], or (iii) high asymmetry between contralesional and ipsilesional CST FA [11]. Past work has also demonstrated that individuals with severe motor impairment and poor integrity of the CST achieve less meaningful motor recovery in the chronic phase [12]. However, there is emerging evidence that data characterizing the CST alone cannot fully explain the spectrum of outcomes that individuals with severe motor impairment after stroke may experience [13–15]. While a meta-analysis of individuals with severe upper limb impairment demonstrated that the presence of a MEP was associated with significantly less arm impairment (higher Fugl-Meyer upper limb score, FM-UL) [7], similar FM-UL scores were noted in people with and without a MEP. Collectively, this suggests that other structures may contribute to motor outcome after stroke.

Focus on the CST as the only biomarker of upper limb motor outcome post stroke neglects the fact that the motor system operates as a network [16, 17]. Research suggests that remote regions in the motor network may be biomarkers of motor outcome in people with severe upper limb impairment [7, 10, 18–21]. There is evidence that the corpus callosum structure (e.g., indexed using DW-MRI) and function (e.g., indexed using TMS) contribute to motor outcome in the chronic phase post stroke. This work suggests that the prefrontal corpus callosum structure (DW-MRI) and function (transcallosal inhibition using TMS) may be a compensatory network to support paretic upper limb movement [10, 22–24].

To date, no work has investigated the combined contribution of the CST and corpus callosum to motor outcome in a cohort of individuals with severe upper limb impairment. The first aim of this cross-sectional study was to define the contribution of the CST and corpus callosum, using measures of both function (from TMS) and structure (from DWI) to upper limb impairment and function in individuals with chronic and severe stroke (FM-UL ≤ 30/66). The second aim was to determine if the biomarker(s) identified in our first aim could distinguish motor outcome subgroups within a group of people with clinically severe upper limb impairment. These findings were compared to a control group who had mild–moderate arm impairment (FM-UL > 40/66). Based on past work [10, 11], we hypothesised that indices of the brain structure (DWI) would explain the greatest variance in motor function and impairment in people with severe motor impairment. Further, we expected that identified brain biomarkers would be unique to the severe group as compared to the mild–moderate group.

## 2. Materials and Methods

Twenty-nine adults with severe (*n* = 15) or mild–moderate (*n* = 14) upper limb impairment after stroke were studied. All were in the chronic phase post stroke (>6 months) [25]. Participants were recruited by convenience sampling from the community and local postings. Inclusion criteria were (1) clinically diagnosed first middle cerebral artery stroke on MRI, (2) residual hemiparesis involving the upper limb, and (3) greater than 12 months post stroke. Exclusion criteria were (1) age < 18 or >85 years, (2) contraindication to TMS or MRI, (3) unable to follow yes/no commands, (4) concomitant neurological or psychiatric disease (beyond stroke), or (5) musculoskeletal disorder interfering with upper limb motor assessment. Ethical approval was received from University of British Columbia and all participants provided written informed consent in accordance with the Declaration of Helsinki.

All participants underwent two assessment sessions: (1) clinical and neurophysiological (TMS) testing and (2) neuroimaging (3T MRI) at the University of British Columbia MRI Research Centre, Vancouver, British Columbia, Canada. In addition, demographic and stroke characteristics were collected from the participants, including stroke date and age at stroke onset.

### 2.1. Clinical Assessment

Valid and reliable tests of upper limb impairment and function were administered by licensed physical therapists that were independent of neuroimaging and neurophysiological assessment collection and analysis. All examiners were trained in the collection of these measures; inter-rater accuracy was confirmed to be >90% by an experienced examiner. The FM-UL was performed to index upper limb impairment. It consists of 33 items rated on a scale from 0 to 2, totalling a possible 66 points [26], where higher scores indicate less motor impairment. We defined participants with a FM-UL score of ≤30 as having severe upper limb impairment [27]. The Wolf Motor Function Test rate (WMFT-rate) [28] indexed upper limb function. It is a valid and reliable measure that has been validated across individuals with a range of upper limb impairment [29]. The test consists of 15 timed movement tasks using the paretic upper limb. Movement time for each task was used to calculate the task rate (WMFT-rate): 60 seconds/time to complete task (in seconds). If an individual could not perform the task in 120 seconds, a mean rate of 0 was given for that task. The average rate of function was calculated across all tasks; faster rates indicate better function [30].

### 2.2. Transcranial Magnetic Stimulation (TMS) Assessment

All TMS sessions were completed with the participant seated comfortably in a height-adjustable chair. TMS was delivered using a figure-of-eight-shaped coil (Magstim 70 mm P/N 9790, Magstim Co., UK) connected to a Magstim 200^2^ stimulator (Magstim Co., UK). The anatomical T_1_ scan for each individual was coregistered to digitized landmarks to enable integration of coil and participant brain anatomy data using the Brainsight™ software package (Rogue Research Inc.), and thus real-time position monitoring. Electromyography (EMG) was collected bilaterally from the participant's extensor carpi radialis (ECR) muscle with 3 cm-diameter circular surface recording electrodes (Covidien, Mansfield, MA) to index TMS-elicited MEPs and ongoing muscle activity. EMG data were collected using LabChart software (LabChart 7.0) and sampled at 2000 Hz, preamplified (1000x) and bandpass filtered at 10–1000 Hz using PowerLab data acquisition system and two bioamplifiers (AD instruments, Colorado Springs, CO). Data were recorded in a 450 ms sweep from 100 ms before to 350 ms after TMS delivery. The “hotspot” for eliciting MEPs in the contralateral extensor carpi radialis (ECR) was found by positioning the coil over the scalp region overlying the hand/forearm representation within the M1 [31]. Standard procedures for determining resting motor threshold (RMT) were performed [32]. TMS pulses were delivered at a random rate between 0.15 and 0.2 Hz during MEP and TCI assessment. When no ipsilesional hotspot was identified (*n* = 13), the location for stimulation was inferred from either (1) the mirrored location of the contralesional hemisphere hotspot or (2) the location of the hand knob identified on the anatomical T_1_ scan.

For TCI assessment, participants were asked to squeeze a handgrip dynamometer (ADInstruments, Colorado Springs, CO) to produce an active isometric contraction in the arm ipsilateral to the identified ECR hotspot. The force signal was digitized and presented on a computer screen in front of the participant for real-time feedback to maintain a constant level of force production during testing. Twelve single TMS pulses were delivered at 150% RMT over the ECR hotspot while participants maintained a unilateral background muscle contraction of 50% maximum grip force output with the ipsilateral hand. When no ipsilesional MEP was identified, TMS pulses were delivered at 80% maximum stimulator output. A custom MATLAB script (MathWorks, Natick, MA) was used to identify the transient reduction in volitional EMG activity elicited by TMS applied over the M1 ipsilateral to the active muscle (termed the ipsilateral silent period or iSP) for each participant.

### 2.3. Processing Ipsilateral Silent Period (iSP)

**Figure 1:**
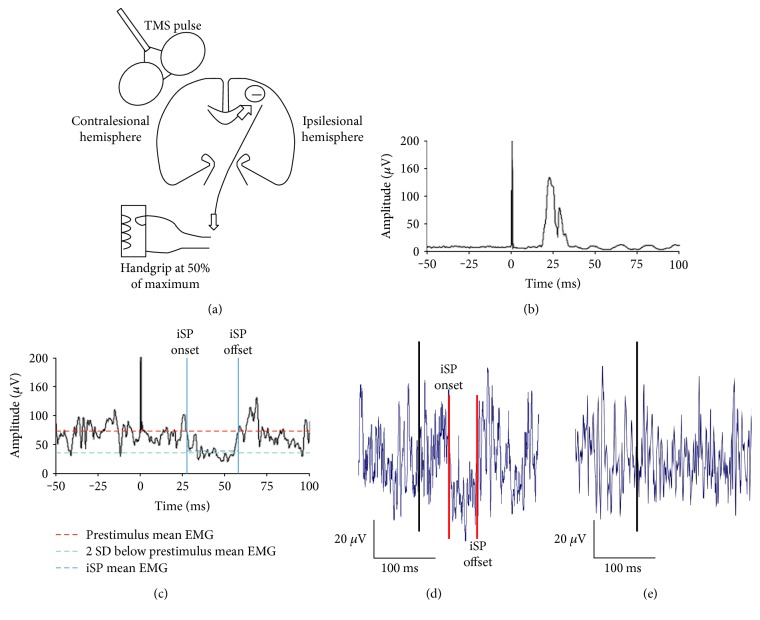
TMS-evoked TCI. (a) A schematic diagram of TMS-evoked TCI. Participants maintain a unilateral voluntary background muscle contraction in the arm ipsilateral to the TMS coil. A single TMS pulse is delivered to the motor cortex. The TMS pulse activates transcallosal pathways which transmit an inhibitory signal (─) to the active motor cortex. This elicits a transient quiescence in the background EMG in the active muscle. In the present example, TMS is delivered over the contralesional hemisphere to elicit the contra-iSP. TCI was also evoked with TMS delivered over the ipsilesional hemisphere to elicit the ipsi-iSP. (b) and (c) Rectified EMG data collected during a TMS session from a representative participant with mild to moderate impairment. (b) The motor evoked potential collected from the contralateral ECR muscle during the TCI procedure. (c) The EMG activity and iSP collected simultaneously from the ipsilateral ECR muscles. The iSP_mean_ ratio was calculated as: iSP mean EMG (blue line)/prestimulus mean EMG (red line). For ease of viewing, only 150 ms of the total 450 ms recording window is displayed. (d) and (e) Representative TCI output from the cohort of individuals with severe arm impairment. (d) The output from an individual when TCI was present. (e) The output from an individual when TCI was not present.

To calculate the iSP, EMG data from each hemisphere were full-wave rectified and averaged for each participant. Mean prestimulus EMG amplitude (100 ms prior to TMS delivery) was defined as baseline muscle activity. The onset of the iSP was defined as the poststimulus time point where the rectified EMG signal fell below prestimulus mean EMG and continued to decrease to less than two standard deviations below this level. The iSP offset was defined as the point at which the EMG signal resumed the level of the prestimulus mean activity consistently for a minimum of 2 ms [33]. All data points between the onset and offset comprised the iSP (Figure 1). The magnitude of iSP was defined as the average EMG level during the iSP (iSP_mean_) relative to the mean prestimulus EMG (iSP_mean_/pre-stim_mean_) [34]. Smaller ratio values indicate larger iSP magnitude and greater TCI generated from the stimulated hemisphere to the contralateral (active) hemisphere. The iSP evoked when TMS was delivered over the contralesional and ipsilesional hemispheres which are termed contralesional-TCI and ipsilesional-TCI, respectively. See Figures 1(a), 1(b), 1(c), 1(d), and 1(e). Two researchers (JN/CM) completed TCI data processing. Twenty-five percent of data were randomly crosschecked; we noted >90% accuracy between personnel.

### 2.4. MRI Acquisition

All MRIs were acquired at the University of British Columbia MRI Research Centre on a Philips Achieva 3.0 T whole-body MRI scanner (Phillips Healthcare, Andover, MD) using an eight-channel sensitivity encoding head coil (SENSE factor = 2.4) and parallel imaging. A high-resolution T1-weighted anatomical scan (TR = 7.47 ms, TE = 3.65 ms, flip angle *θ* = 6°, FOV = 256 × 256 mm, 160 slices, and 1 mm^3^ isotropic voxel) was collected to determine lesion location. A single high-angular resolution diffusion imaging (HARDI) scan was subsequently performed using a single-shot echo planar imaging (EPI) sequence (TR = 7096 ms, TE = 60 ms, FOV = 224 × 224 mm, 70 slices, and voxel dimensions = 2.2 × 2.2 × 2.2 mm^3^). Diffusion weighting was applied across 60 independent noncollinear orientations (*b* = 700 s/mm^2^), along with a single unweighted image (*b* = 0 s/mm^2^).

### 2.5. Preprocessing

DWI data were first visually inspected for excessive motion artifact or instrumental noise using quality assurance tools available in the diffusion MRI software package ExploreDTI v4.2.2 (http://www.exploredti.com) [35]. For all images, signal intensity was modulated and the b-matrix rotated [36]. Imaging data were then corrected for motion and distortion with images in native space for corpus callosum (CC) and corticospinal (CST) tractography. Constrained spherical deconvolution (CSD) was used to model diffusion behaviour [37]. CSD was chosen as it is robust in the presence of multiple fibre populations (estimated to occur in greater than 90% of white matter voxels in the brain [38]) as it does not make assumptions regarding uniform diffusion of water within a voxel [37, 39] and is more sensitive in the severely damaged brain [40]. CSD-based deterministic whole-brain fibre tractography was initiated at each voxel using the following parameters: seedpoint resolution of 2 mm^3^, 0.2 mm step size, maximum turning angle of >40°, and fibre length range of 50–500 mm [41]. Tractography employed a fibre alignment by continuous tracking algorithm approach [42] with FA values extracted from reconstructed streamlines. FA is a quantitative, unit-less measure of diffusion behaviour of water in the brain influenced by microstructural properties of white matter and is the most commonly reported measure of white matter microstructural properties after stroke [43]. All regions of interest were hand drawn by experienced personnel (KH/JN/CM). We have previously established the inter-rater reliability of our processing and analysis procedures [40, 44].

### 2.6. Corticospinal Tract

**Figure 2:**
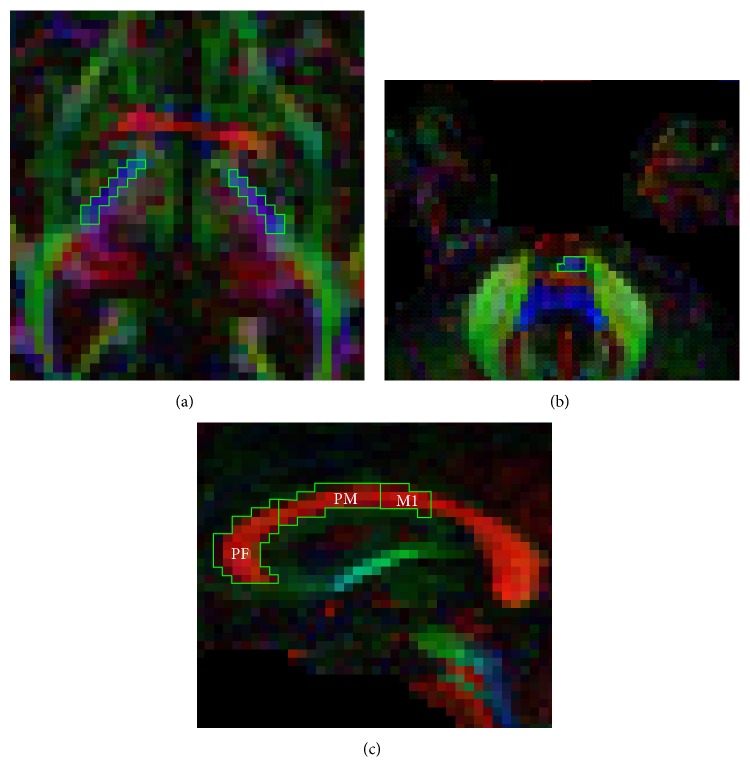
Representative regions of interest drawn on FA MAPS in ExploreDTI of an individual with severe arm impairment. Corticospinal tract regions of interest were drawn in the axial plane at the level of the (a) anterior commissure, and (b) pons as defined by Mang et al. [10]. (c) Corpus callosum regions of interest were drawn in the midsagittal plane according to a geometric partitioning scheme for regions I, II, and III according to Hofer and Frahm [46].

Two regions of interest (ROIs) were drawn and applied to extract streamlines from the entire length of the CST. Firstly, a ROI was delineated in each hemisphere in the axial plane [45] as a “SEED” ROI around the posterior limb of the internal capsule at the level of the anterior commissure, a region through which motor fibres descend [45]. Secondly, a logical “AND” ROI was constructed in each hemisphere, around the CST at the level of the pons. The “AND” function introduced the requirement that only fibres passing through both the “SEED” and “AND” ROIs would be included for fibre tracking, similar to previous work [10]. On the basis of these ROIs, subsequent tract reconstructions of descending CST streamlines were produced and mean FA was calculated across the entire length of the reconstruction. To compute the asymmetry index (equation: contralesional CST FA − ipsilesional CST FA/contralesional CST FA + ipsilesional CST FA). Figures 2(a) and 2(b) illustrate the CST ROIs in ExploreDTI.

### 2.7. Corpus Callosum

Callosal subregion ROIs were manually delineated in the midsagittal plane according to a geometric partitioning scheme [46]. Based on previous findings [10] and anatomical relevance [46], for this analysis, only streamlines projecting to subregion I (prefrontal, PF-CC), subregion II (premotor, PM-CC), and subregion III (primary motor, M1-CC) were extracted. Using each subregion ROI separately as a “SEED,” interhemispheric streamlines passing these subregions of the corpus callosum were isolated and mean FA was calculated across the entire length of the reconstructed streamlines.  Figure 2(c) illustrates the CC ROIs in Explore DTI.

### 2.8. Statistical Analysis

The primary dependent variable of interest was paretic WMFT-rate and the secondary dependent variable of interest was paretic FM-UL. Spearman's correlation coefficients (rho) were determined for the following variables in individuals with severe impairment: age, months since stroke onset, ipsilesional MEP (yes/no), ipsilesional-TCI, contralesional-TCI, ipsilesional CST FA, contralesional CST FA, PF-CC FA, PM-CC FA, and M1-CC FA. All variables with a *p* < 0.05 were considered to be significantly correlated with each dependent measure, while all variables with a *p* < 0.1 were considered to have a trending correlation with each dependent measure. A stepwise regression model was then performed for each dependent measure in individuals with severe impairment; all variables entered were at least *p* < 0.1. Separate stepwise regression models with these variables were performed for individuals with severe impairment and individuals with mild–moderate impairment. The stepping criteria was *p* < 0.05 to add and *p* > 0.1 to remove variables. We did not correct for multiple comparisons on the principle that the restrictiveness of Bonferroni correction could hinder initial exploratory studies with low participant numbers [47–49]. All statistics were performed in SPSS v23.0.

To determine if a variable identified in the stepwise regression model was able to differentiate levels of impairment and function within the severely impaired group, we used a two-group k-means cluster analysis. This approach provides a hypothesis-free classification of participants according to a variable of interest. It was performed when a single variable in isolation explained a significant amount of the variance in impairment or function. The mean difference between clusters was used to define higher and lower motor outcome clusters. To confirm that clusters differentiated meaningful subgroups based on model allocation, we investigated if clusters were separated by a minimal clinically important difference in motor impairment or function. For impairment, mean FM-UL scores for identified clusters should be separated by at least 5.75 points [50], and for motor function, mean WMFT-rate scores for identified clusters should be separated by >10% difference between groups [51]. Independent sample *t*-tests indicated descriptively the cluster analyses' goodness-of-fit (*p* < 0.05). Based on cluster model allocation, specificity and sensitivity of the algorithm for impairment and function outcomes were determined.

## 3. Results

Fifteen individuals with chronic, severe upper limb impairment (FM-UL mean 17 ± 7) and 14 individuals with chronic, mild–moderate upper limb impairment (FM-UL mean 58 ± 4) were evaluated.  Table 1 outlines the group summary demographic, motor impairment and function outcomes, and Table 2 contains information on dependent and independent variables for each individual participant.

### 3.1. Upper Limb Function (WMFT-Rate)

**Figure 3:**
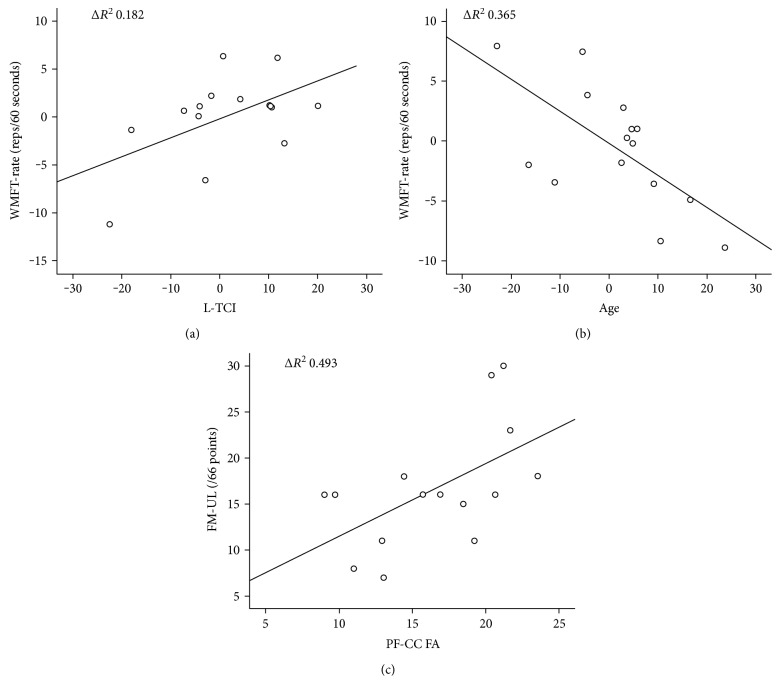
Partial plots for stepwise multiple linear regression model for the cohort of individuals with severe upper limb impairment, *n* = 15. (a) Wolf Motor Function Test rate and transcallosal inhibition from ipsilesional M1 to contralesional M1; (b) Wolf Motor Function Test rate and age; and (c) Fugl-Meyer upper limb assessment, prefrontal corpus callosum. WMFT-rate: Wolf Motor Function Test rate; L-TCI: lesioned transcallosal inhibition; FM-UL: Fugl-Meyer upper limb; PF-CC FA: prefrontal corpus callosum fractional anisotropy.

Age (rho = −0.682, *p* = 0.005) and PF-CC (rho = 0.609, *p* = 0.016) were significantly associated with paretic WMFT-rate, while contralesional-CST (rho = 0.486, *p* = 0.066) and ipsilesional-TCI (rho = 0.447, *p* = 0.095) showed a trend towards significance. The stepwise regression model identified age (*R*^2^ change 0.365, *p* = 0.017) and ipsilesional-TCI (*R*^2^ change 0.182, *p* = 0.048) to explain 54.7% (*p* = 0.009) of the variance in paretic WMFT-rate (Figures 3(a) and 3(b)). The same model was performed in individuals with mild–moderate upper limb impairment and no variables emerged to significantly explain variance of paretic WMFT-rate in this group (*p* > 0.05).

### 3.2. Upper Limb Impairment (FM-UL)

PF-CC (rho = 0.658, *p* = 0.008), PM-CC (rho = 0.747, *p* = 0.001), and M1-CC (rho = 0.715, *p* = 0.003) were significantly associated with FM-UL. The stepwise regression model identified PF-CC as significantly explaining 49.3% of the variance in FM-UL (*p* = 0.007) (Figure 3(c)). The same model was performed in a cohort of individuals with mild–moderate upper limb impairment and no variables emerged to significantly explain the variance of the paretic FM-UL (*p* > 0.05).

### 3.3. Cluster Analysis

**Figure 4:**
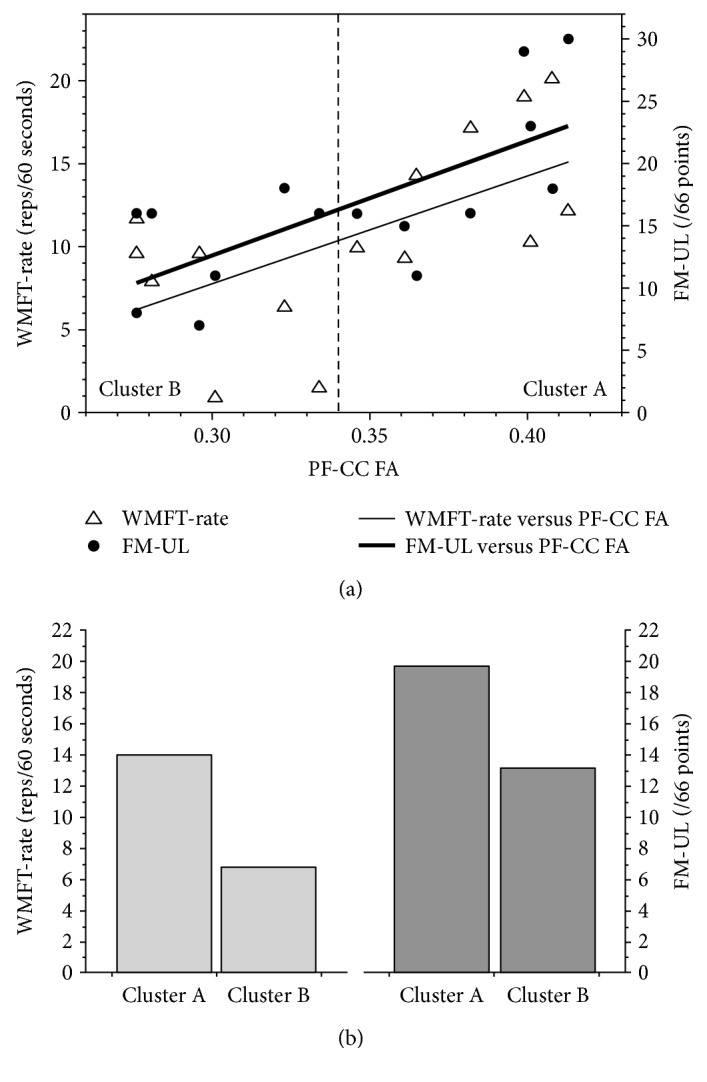
(a) From the group of individuals with severe arm impairment (*n* = 15), k-means cluster analysis of PF-CC FA identified two groups: cluster A, higher outcome, and cluster B, lower outcome. (b) Between group differences for cluster A and cluster B were significant for function (Wolf Motor Function Test rate (WMFT-rate), *p* = 0.006) and impairment (Fugl-Meyer upper limb (FM-UL), *p* = 0.049). PF-CC: prefrontal corpus callosum; FA: fractional anisotropy; WMFT-rate: Wolf Motor Function Test rate; FM-UL: Fugl-Meyer upper limb.

k-means cluster analysis was performed using PF-CC FA. Two clusters were identified: cluster A mean PF-CC FA = 0.384 and cluster B mean PF-CC FA = 0.298. The midpoint between clusters was 0.341 (Figure 4(a)), with modeling allocating individuals with a PF-CC FA above the midpoint labeled as cluster A, higher outcome, and PF-CC FA below the midpoint labeled as cluster B, lower outcome. The clusters represented clinically relevant groups (Figure 4(b)), demonstrated by clinically meaningful and significant differences between groups for motor impairment (FM-UL mean difference 6.6 points, *t* = 2.174, df 13, *p* = 0.049) and motor function (WMFT-rate mean difference 7.3 repetitions, *t* = 3.316, df 13, *p* = 0.006). Based on this clustering, specificity was 43%, but sensitivity was 88% for FM-UL. For WMFT-rate, specificity was 86% and sensitivity was 88%.

## 4. Discussion

Ipsilesional TCI from M1 (function) and prefrontal corpus callosum (structure) explained significant variance in motor outcome at a single chronic time point in individuals with severe upper limb impairment post stroke. Contrary to our hypothesis, a structural index derived from DWI did not explain greatest variance in upper limb function. Rather, interhemispheric inhibition indexed from the ipsilesional to contralesional M1 with TCI, along with younger age, explained the most variance in motor function. An index of brain structure (PF-CC FA) did explain the greatest amount of variance in arm impairment, and this variable was associated with function. Building upon these findings, a cluster analysis using PF-CC FA identified two clusters that were separated by a clinically meaningful and significant difference in motor impairment and function. While it is unlikely that one variable will accurately prognosticate all individuals, it appears that interhemispheric communication is an important consideration to understand motor outcomes in the chronic phase.

### 4.1. Prefrontal Corpus Callosum Structure Differentiated Motor Outcome Experienced by People with Severe Upper Limb Impairment

Structural integrity of the prefrontal segment of the corpus callosum (PF-CC) explained motor outcome over and above ipsilesional corticospinal tract integrity in people with chronic and severe upper limb impairment. Given that the CST had sustained significant damage, coupled with a higher probability of prefrontal areas surviving after stroke [52], it is perhaps not surprising that structures in the prefrontal region would emerge as the principal explanatory variable. Failure of CST streamlines alone to be related to better outcome in individuals with severe stroke is consistent with our recent individual patient data review [7] and other empirical work [14, 21]. Importantly, PF-CC structural integrity differentiated individuals with chronic and severe upper limb impairment after stroke into two subgroups that were separated by a clinically meaningful difference in motor impairment and function outcome scores. This identifies a biological state in the chronic phase that could be tested in acute stroke trials in the future. Such data would enable evaluation of whether structural integrity in the PF-CC is critical in the acute stage or if it reflects a compensatory pattern that emerges with time post stroke.

### 4.2. How May the Prefrontal Corpus Callosum Contribute to Better Outcome after Severe Stroke?

The emergence of the prefrontal corpus callosum may represent a compensatory pathway that can mediate motor outcomes in people with chronic and severe upper limb impairment after stroke. Building on evidence from the current study and previous studies of humans with [22] and without stroke [10], as well as nonhuman primates [53, 54], we propose a theoretical framework that underpins our findings.

Firstly, prefrontal regions of the brain are remotely connected to M1. A study of rhesus monkeys demonstrated that prefrontal regions of the brain (including regions 46, 9, 46v, and 46d) have projections to the premotor cortex (regions 6d, 6v) and subsequently the primary motor cortex (region 4) [54]. In humans, we cannot establish this multistep pathway. However, inhibitory signals are able to be sent from dorsolateral prefrontal cortex to M1 (within hemisphere) and between hemispheres, that is, the ipsilateral dorsolateral prefrontal cortex to the contralateral M1 in healthy young and healthy older individuals [55], as well as people with mild to severe arm impairment after stroke [56]. Therefore, it is possible that a pathway originating in the prefrontal area and ending in the primary motor area exists to be exploited.

Secondly, signals from the primary motor cortex can descend peripherally to the ipsilateral upper limb. Studies of CST architecture demonstrate that 10–15% of CST fibres do not cross the midline in the brainstem and form the anterior CST [57]. Tracking these fibres peripherally indicates that they remain ipsilateral to innervate the proximal arm musculature. Individuals post stroke that exhibit little sparing of the ipsilesional CST may benefit from tapping into this anatomical redundancy as it may be the only remaining means by which paretic motor output may be produced [13]. Indeed, proximal movements are more likely to return to the paretic upper limb after severe stroke [4, 58, 59]. Our data show that higher FA from the contralateral CST (indexed with DW-MRI) was associated with higher motor function, suggesting that this pathway may be used to enable motor outcomes in the paretic limb of those with severe motor impairment after stroke.

Thirdly, less inhibition from the ipsilesional to contralesional M1, which was associated with better motor function (higher WMFT-rate) in people with severe impairment, could also support access to uncrossed, anterior CST fibres. This fits with previous work which found that suppression of contralesional M1 activity, through interventions such as inhibitory repetitive TMS, may be contraindicated for individuals with major disruption of the ipsilesional CST [60].

Taken together, interhemispheric communication (structural PF-CC indexed using DW-MRI and functional TCI indexed from TMS) may support a compensatory mechanism that helps to overcome poor ipsilesional CST integrity. Our findings provide a preliminary framework to test in a nonhuman primate model and in longitudinal studies of humans with severe upper limb impairment post stroke. If found to be reliable, they may provide a potential cortical target for noninvasive brain stimulation techniques, such as repetitive TMS or transcranial direct current stimulation, that attempt to amplify functionally relevant brain regions.

### 4.3. Strengths and Limitations

This study represents an important advance in our understanding of the biological state of the brain in the chronic stage in a cohort of individuals with severe upper limb impairment. However, it has several limitations. Firstly, our design is cross-sectional and conducted in the chronic phase post stroke. We did not have access to information about participant status early post stroke. As such, it does not enable us to understand if our finding is a recovery-mediated outcome that reflects the system's best effort to respond to motor limitations. A longitudinal design that recruits participants early post stroke (e.g., within the first 7–28 days post stroke) and follows participants at meaningful time points (e.g., 3–6 and 12 months post stroke) is required to answer this question [25]. Secondly, we performed a k-means cluster on the same small cohort of severe stroke participants to confirm the findings of the regression analysis. This provided capacity to confirm that there were two groups in the current data based on PF-CC FA, but our data cannot be used to confirm a cut point or for individual prognostication. A larger, independent sample with longitudinal data from multiple sites is required to achieve this objective. Thirdly, the streamlines evaluated are reconstructions; they do not definitively establish whether structural connections provide afferent or efferent input, to or from a target region. As such, we only hypothesise the potential cascade of signals from the information collated. Fourth, our collection of TCI involved a handgrip contraction that generated EMG activity in both wrist extensors and flexors, which may have resulted in an underestimation of iSP due to reciprocal inhibition between the flexors and extensors in the nervous system. However, the individuals in this study had severe upper limb impairment and were unable to perform isolated wrist extension; therefore, whole handgrip contraction was employed. Finally, as is the case for all diffusion imaging studies, the number of streamlines extracted is based on reconstruction of imaging data derived from an algorithm computed with the software (*Explore DTI 4.2.2*) and errors related to midline shift, physiological noise, and microstructural changes in brain tissue may have influenced microstructural properties extracted. These limitations may collectively increase the risk of type 1 bias.

## 5. Conclusion

The data in this study suggest that the interhemispheric communication may support motor outcomes in individuals with severe and chronic upper limb impairment after stroke. The prefrontal areas could be a target for interventional studies that attempt to enable motor recovery of people with severe upper limb impairment. Further investigation is required to determine the role of PF-CC early post stroke and in mediating recovery.

## Figures and Tables

**Table 1:** Summary of group demographics and stroke characteristics.

	*n* = 15 severe	*n* = 14 mild–moderate
Age, year, mean (SD)	58.3 ± 12	68.3 ± 9.2
Gender, *n* = male : female	8 : 7	11 : 3
Lesion location, *n* = subcortical : cortical	9 : 6	11 : 3
Affected hemisphere, *n* = left : right	8 : 7	6 : 9
Affected arm, *n* = left : right	7 : 8	9 : 6
Months post stroke, mean (SD)	61.6 ± 50.0	80.1 ± 70.6
Fugl-Meyer upper limb, mean (SD), /66	16.7 ± 6.6	58.3 ± 4.0
Wolf Motor Function Test, rate, mean (SD)	10.6 ± 5.5	42.2 ± 12.0

**Table 2:** Individual participant demographics and stroke characteristics.

ID	Stroke location	MSS	Age	FM-UL	WMFT-rate, NP	WMFT-rate, P	L MEP Rest	L TCI mean	NL TCI mean	PF-CC FA	PM-CC FA	M1-CC FA	L CST FA	NL CST FA
1	Subcortical	41	63	23	71.5	10.3	0	0.64	0.66	0.40	0.40	0.41	0.39	0.52
2	Cortical	91	61	16	83.0	11.7	0	0.73	0.74	0.23	0.13	0.20	0.31	0.49
3	Cortical	85	62	8	53.9	9.6	0	0.64	0.84	0.28	0.25	0.30	0.36	0.50
4	Cortical	94	57	7	50.6	9.6	0	0.52	—	0.30	0.29	0.34	0.35	0.45
5	Cortical	22	51	16	50.6	1.5	0	0.49	0.80	0.33	0.36	0.42	0.46	0.51
6	Cortical	25	69	11	46.6	0.9	0	0.64	0.81	0.30	0.29	0.30	0.33	0.50
7	Subcortical	21	65	15	45.7	9.3	0	0.60	0.80	0.36	0.35	0.35	0.37	0.46
8	Subcortical	22	57	16	75.9	17.1	0	0.82	0.83	0.38	0.40	0.41	0.45	0.53
9	Subcortical	94	36	11	61.0	14.3	0	0.88	0.80	0.37	0.29	0.22	0.26	0.49
10	Subcortical	145	64	16	62.8	9.9	1	0.79	0.91	0.35	0.36	0.43	0.47	0.50
11	Subcortical	23	72	16	71.8	7.9	0	0.76	0.93	0.28	0.36	0.38	0.45	0.42
12	Subcortical	33	33	18	146.8	20.1	0	0.74	—	0.41	0.42	0.42	0.33	0.55
13	Subcortical	47	51	29	62.3	19.0	0	0.72	0.77	0.40	0.41	0.43	0.44	0.58
14	Cortical	160	57	30	27.4	12.1	1	0.80	0.89	0.41	0.41	0.45	0.43	0.47
15	Subcortical	21	77	18	32.6	6.4	0	0.85	0.59	0.32	0.32	0.37	0.40	0.49

Mean	—	61.6	58.3	16.7	62.8	10.7	—	0.71	0.80	0.34	0.33	0.36	0.39	0.50
SD	—	47.0	12.02	6.6	27.9	5.5	—	0.12	0.10	0.05	0.08	0.08	0.06	0.04
Min	—	21.0	33.0	7.0	27.4	0.9	—	0.49	0.59	0.28	0.13	0.20	0.26	0.42
Max	—	160.0	77.0	30.0	146.8	20.1	—	0.88	0.93	0.41	0.42	0.45	0.47	0.58

1	Subcortical	155	76	49	48.17	34.11	1	1.00	0.68	0.32	0.29	0.36	0.39	0.52
2	Subcortical	23	60	54	54.77	39.90	1	0.62	0.67	0.32	0.32	0.42	0.51	0.51
3	Cortical	270	59	55	44.71	23.98	1	0.56	0.74	0.38	0.29	0.33	0.38	0.51
4	Subcortical	83	71	56	64.21	52.01	1	0.65	0.53	0.37	0.38	0.42	0.41	0.44
5	Cortical	94	64	56	55.27	46.88	1	1.00	0.67	0.36	0.22	0.13	0.42	0.48
6	Subcortical	15	69	57	62.82	52.99	1	0.69	0.63	0.40	0.41	0.45	0.45	0.53
7	Subcortical	82	67	59	79.77	62.95	1	0.65	0.67	0.34	0.33	0.40	0.46	0.52
8	Subcortical	142	73	60	76.42	57.58	1	0.57	0.61	0.40	0.37	0.40	0.39	0.49
9	Subcortical	35	85	60	40.72	34.49	1	0.44	0.69	0.34	0.31	0.35	0.41	0.49
10	Subcortical	18	79	61	54.93	45.00	1	1.00	1.00	0.33	0.33	0.40	0.45	0.49
11	Subcortical	81	76	62	63.57	64.07	1	0.78	0.82	0.37	0.36	0.41	0.45	0.41
12	Cortical	67	65	62	52.35	44.44	1	1.00	0.52	0.35	0.37	0.35	0.35	0.48
13	Subcortical	20	62	62	64.96	58.65	1	0.68	1.00	0.37	—	0.44	0.44	0.43
14	Subcortical	37	50	63	61.49	58.16	1	0.62	0.55	0.38	0.39	0.38	0.47	0.49

Mean	—	80.0	68.0	58.3	58.9	48.2	—	0.73	0.69	0.36	0.34	0.37	0.41	0.49
SD	—	71.0	9.2	4.0	11.1	12.0	—	0.19	0.15	0.03	0.05	0.08	0.06	0.03
Min	—	15.0	50.0	49.0	40.7	24.0	—	0.44	0.52	0.32	0.22	0.13	0.31	0.43
Max	—	270.0	85.0	63.0	79.8	64.1	—	1.00	1.00	0.40	0.41	0.45	0.51	0.53

CST: corticospinal tract; FA: fractional anisotropy; FM-UL: Fugl-Meyer upper limb; M1-CC: primary motor corpus callosum; min: minimum; max: maximum; MSS: months since stroke; L: ipsilesional; NL: contralesional; NP: nonparetic; P: paretic; PF-CC: prefrontal corpus callosum; PM-CC: premotor corpus callosum; SD: standard deviation; WMFT: Wolf Motor Function Test rate.
